# Multimodal fMRI Resting-State Functional Connectivity in *Granulin* Mutations: The Case of Fronto-Parietal Dementia

**DOI:** 10.1371/journal.pone.0106500

**Published:** 2014-09-04

**Authors:** Enrico Premi, Franco Cauda, Roberto Gasparotti, Matteo Diano, Silvana Archetti, Alessandro Padovani, Barbara Borroni

**Affiliations:** 1 Centre for Ageing Brain and Neurodegenerative Disorders, Neurology Unit, University of Brescia, Brescia, Italy; 2 Clinical and Experimental Center for Functional Magnetic Resonance Imaging, Koelliker Hospital, Turin, Italy; 3 Department of Psychology, University of Turin, Turin, Italy; 4 Neuroradiology Unit, University of Brescia, Brescia, Italy; 5 III Laboratory Analyses, Brescia Hospital, Brescia, Italy; Hangzhou Normal University, China

## Abstract

**Background:**

Monogenic dementias represent a great opportunity to trace disease progression from preclinical to symptomatic stages. Frontotemporal Dementia related to Granulin (*GRN*) mutations presents a specific framework of brain damage, involving fronto-temporal regions and long inter-hemispheric white matter bundles. Multimodal resting-state functional MRI (rs-fMRI) is a promising tool to carefully describe disease signature from the earliest disease phase.

**Objective:**

To define local connectivity alterations in *GRN* related pathology moving from the presymptomatic (asymptomatic *GRN* mutation carriers) to the clinical phase of the disease (*GRN*- related Frontotemporal Dementia).

**Methods:**

Thirty-one *GRN Thr272fs* mutation carriers (14 patients with Frontotemporal Dementia and 17 asymptomatic carriers) and 38 healthy controls were recruited. Local connectivity measures (Regional Homogeneity (ReHo), Fractional Amplitude of Low Frequency Fluctuation (fALFF) and Degree Centrality (DC)) were computed, considering age and gender as nuisance variables as well as the influence of voxel-level gray matter atrophy.

**Results:**

Asymptomatic *GRN* carriers had selective reduced ReHo in the left parietal region and increased ReHo in frontal regions compared to healthy controls. Considering Frontotemporal Dementia patients, all measures (ReHo, fALFF and DC) were reduced in inferior parietal, frontal lobes and posterior cingulate cortex. Considering *GRN* mutation carriers, an inverse correlation with age in the posterior cingulate cortex, inferior parietal lobule and orbitofrontal cortex was found.

**Conclusions:**

*GRN* pathology is characterized by functional brain network alterations even decades before the clinical onset; they involve the parietal region primarily and then spread to the anterior regions of the brain, supporting the concept of molecular nexopathies.

## Introduction

Frontotemporal Lobar Degeneration (FTLD) is a neurodegenerative disorder characterized by behavioural abnormalities, language impairment, and deficits in executive functions as the most typical clinical features [1]–[4]. FTLD is clinically heterogeneous, as different prototypical variants have been carefully described. On the basis of presenting clinical symptoms, behavioural variant FTD (bvFTD), agrammatic variant of Primary Progressive Aphasia (avPPA), and semantic variant of PPA (svPPA) represent the most common phenotypes [3], [4]. Each one presents specific neuroimaging hallmarks; bvFTD is characterised by mesial and dorsolateral frontal damage, prevalent on the right side, avPPA is defined by involvement of Broca's area and left insula, whilst svPPA usually presents left rostral temporal involvement [5]–[7].

If clinical pictures are highly correlated with established patterns of neuroimaging features, on the other hand, no definite correlation between clinical and pathogenetic mechanisms may be assessed. Neuropathological classification of Frontotemporal Dementia (FTD) is based on the major constituents of the cellular inclusions present, such as tau, TAR-DNA-binding protein-43 (TDP-43) or fused-in-sarcoma (FUS) protein, designated FTLD-tau, FTLD-TDP or FTLD-FUS respectively [8]. Moreover, a number of genes have been recognized as causative of autosomal dominant inherited disorder, such as mutations within *Microtuble Associated Protein Tau (MAPT)* and *Granulin (GRN)* genes along with repeat expansion of *C9orf72* gene [9], [10]. Genetic trait seems to be characterized by a specific framework of brain damage; in particular, *GRN* patients usually had more asymmetric fronto-temporo-parietal atrophy [11]–[13], as well as a more severe impairment of Salience Network connectivity [14] and reduction of effective connectivity in temporo-parietal regions [15] Furthermore, considering asymptomatic *GRN* carriers, an altered resting-state functional connectivity in anterior and posterior regions was evident [13], [16]-[19]. Recently, we explored resting state fMRI connectivity with ICA approach in asymptomatic *GRN* carriers, showing a reduced connectivity in left Frontoparietal Network and an increased connectivity in Executive Network compared to healthy controls [16].

Genetic mutations clearly mirror the underpinnings of the disease, and give the opportunity to assess its pathogenetic and biological mechanism. Among others, *GRN* mutations are expected to induce a loss of 50% of progranulin, with a mechanism of haploinsufficiency, and the presence of ubiquitinated TDP-43 protein is the neuropathological hallmark [8], [20], [21]. The physiological role of progranulin, as well as the effect of its reduction in the brain, is still largely unknown, although it has been recently suggested that progranulin might be involved in inflammatory pathways and innate immunity [22], [23], and that it acts as a neurotrophic factor [24]. Improved understanding of the molecular mechanisms of network disintegration will constitute a new paradigm of neurodegenerative disease and monogenic dementias.

Neuroimaging techniques, such as functional resting state MRI, represent an *in-vivo* non invasive tool to study variable intrinsic brain vulnerability and to follow disease progression from the preclinical phase to symptomatic stages. In the last few years, a number of functional parameters have been introduced to study brain functional network abnormalities. Regional Homogeneity (ReHo) [25] look at the coherence of focal brain spontaneous low-frequency (<0.08 Hz) BOLD signal fluctuations at whole-brain level. Hemodynamic characteristics of every voxel in a functional cluster would be similar to the neighbor voxels [25], [26], and thus allows the mapping of the whole-brain regional activity [27]–[29]. The fractional amplitude of low frequency fluctuation (fALFF), describes the power of the signal in the low frequency range (0.01–0.08 Hz), as an index of local power of BOLD signal [30], [31]. Finally, Degree Centrality (DC) allows the study of the nodes that form the whole-brain network (also known as functional connectome), and in particular the nodes that are considered “central” within the network [32]–[34]; DC is an index of local connectivity by counting the number of direct connections from one node (voxel) to all other nodes. Higher DC values indicates key-nodes for the network, and are crucial for the maintenance of the whole functional network. With regard to brain networks, this index could correlate with highly associative cortical areas reflecting the intrinsic cortical organization (Hub architecture). Damage of one area with high DC leads to more serious damage in the inter-neuronal connection and to an increase in the erroneous use of information in reaching the target. Rather than a group of separate functional brain networks, brain connectome is based on different inter-related modules that cooperate to achieve cognitive functioning [35]–[37]. Indeed, it has become clear that brain diseases are not strictly related to a single network dysfunction but multimodal damage can occurs [38].

Elucidating how pathogenic molecules produce specific brain network disintegration may contribute to the creation of a new nomenclature of FTD, based on pathogenetic mechanisms. With this in mind, in the present work we studied a group of subjects carrying *GRN Thr272fs* mutation (FTD patients and still asymptomatic subjects in order to evaluate: 1) the functional connectivity alterations in the different disease stages, from preclinical to symptomatic phases; 2) the effect of age on *GRN* related pathology, in order to trace progressive brain damage during disease course.

## Methods

### Subjects

Subjects participating in the present study were recruited from the Centre for Ageing Brain and Neurodegenerative Disorders, at University of Brescia (Brescia, Italy). The studied sample included 14 patients with FTD carrying *GRN Thr272fs* mutation (*GRN+*), and 16 age and gender-matched healthy subjects (HC). Furthermore, 17 asymptomatic carriers of *GRN Thr272fs* mutation (*aGRN+*) and 22 non-carriers belonging to the same families (young healthy controls, yHC) were recruited. FTD patients met current clinical diagnostic criteria either for bvFTD (7 cases) or avPPA (7 cases) [3], [4]. An extensive neuropsychological assessment in both patients and asymptomatic siblings, including the FTD-modified Clinical Dementia Rating scale (FTD-modified CDR) was administered, as described elsewhere [39]. Written informed consent (from the subject or from the responsible guardian if the subject was incapable, as demonstrated by clinical and neuropsychological evaluation, showing functional impairment in the activity of daily living) was obtained, for each procedure, before study initiation, as well as blood collection byvenous puncture, genetic analysis, and MRI scanning. The research protocol was approved by the ethics committee of the Brescia Hospital. The work conformed to the Helsinki Declaration.

### Granulin sequencing

Genomic DNA was extracted from peripheral blood using a standard procedure. All the 12 exons plus exon 0 of *GRN*, and at least 30 base pairs (bp) of their flanking introns were evaluated by polymerase chain reaction (PCR) and subsequent sequencing. *GRN Thr272fs* (*g*.1977_*1980 delCACT*) was tested as described elsewhere [40].

### MRI acquisition

All imaging was obtained using a 1.5T Siemens symphony magnetic resonance scanner (Siemens, Erlangen, Germany), equipped with a circularly polarized transmit-receive coil. In a single session, the following scans were collected from each studied subject: (1) Dual-echo turbo spin echo (TSE) (repetition time [TR] = 2500 ms, echo time [TE] = 50 ms), to exclude the presence of macroscopic brain abnormalities, according to exclusion criteria; (2) 3D magnetization-prepared rapid gradient echo (MPRAGE) T1-weighted scan (TR = 2010 ms, TE = 3.93 ms, matrix = 1×1×1, in-plane field of view [FOV] = 250×250 mm^2^, slice thickness = 1 mm, flip angle = 15°); and (3) T2*-weighted echo planar (EPI) sensitized to blood oxygen level dependent (BOLD) contrast (TR = 2500 ms, TE = 50 ms, 29 axial slices parallel to anterior commisure–posterior commissure line (AC-PC) line, matrix = 64×64, field of view = 224 mm, slice thickness = 3.5 mm) for resting state fMRI. Blood oxygen level dependent EPI images were collected during rest for an 8-minute period, resulting in a total of 195 volumes. During this acquisition, subjects were instructed to keep their eyes closed, not to think of anything in particular, and not to fall asleep.

### MRI preprocessing

All preprocessing steps were carried out using Advanced Data Processing Assistant for Resting-State fMRI (DPARSFA) (http://rfmri.org/DPARSF) which is based on Resting-State fMRI Data Analysis Toolkit (REST, http://www.restfmri.net) [41] and Statistical Parametric Mapping (SPM8) (http://www.fil.ion.ucl.ac.uk/spm). T1-weighted images from all recruited subjects were visually inspected for a qualitative assessment of macroscopic atrophy, and to check for the quality of data before carrying out a quantitative volumetric analysis. For each subject, an iterative combination of segmentations and normalizations (implemented within the “Segment” module in SPM8) produced a GM probability map [42] in Montreal Neurological Institute (MNI) coordinates. To compensate for compression or expansion during warping of images to match the template, GM maps were modulated by multiplying the intensity of each voxel by the local value derived from the deformation field (Jacobian determinants) [43]. All data were then smoothed using a 10 mm FWHM Gaussian kernel. For each subject the first 4 volumes of the fMRI series were discarded to allow for T1 equilibration effects. The remaining 191 volumes were compensated for slice-dependent time shifts, corrected for geometrical displacements according to the estimated head movement and realigned to the first volume. Correction for head motion [44] and head motion scrubbing regressor [45] was also performed. Any subject who had a maximum displacement in any direction larger than 1.5 mm, or a maximum rotation (x,y,z) larger than 1.5° was excluded. All data were subsequently spatially normalized to the T1 unified segmentation template in Montreal Neurological Institute coordinates derived from SPM8 software and resampled to 3×3×3 cubic voxels. A linear regression to remove sources of spurious variances (motion parameters, linear drift and the average time series in the cerebrospinal fluid and white matter regions) was performed. Then, all images were filtered by a phase-insensitive bandpass filter (pass band 0.01–0.08 Hz) to reduce the effect of low frequency drift and high frequency physiological noise. Finally, a spatial smoothing with an isotropic Gaussian kernel (full-width at half-maximum, 8 mm) was applied [25] to reduce spatial noise. This last step was used for all the analyses except for Regional Homogeneity; in fact, previous studies demonstrated that spatial smoothing artificially enhanced the ReHo intensity [28], [46]. For this reason, in this case, the spatial smoothing was carried out after ReHo calculation. All the preprocessing steps to obtain the functional maps below (ReHo, fALFF, DC) were performed with DPARSFA.

### Regional Homogeneity (ReHo)

Regional Homogeneity (ReHo) maps regional activity across the whole brain [25], measuring the degree of regional synchronization of fMRI time courses. Greater ReHo values indicate greater regional synchronization. ReHo was performed on a voxel-by-voxel basis by calculating Kendall's coefficient of concordance [26] of a time series of a given voxel and those of its 26 neighboring voxels within a brain mask (provided by DPARSFA and excluding non-brain areas). In order to reduce the effect of individual variability, a global mean normalization was applied, by dividing ReHo value by the mean ReHo of the whole brain for each subject [25], [28], [46], [47] as ReHo_normalized_ = ReHo(x,y,z)/mean(ReHo). As described above, a spatial smoothing was subsequently applied (FWHM = 8×8×8 mm).

### Fractional Amplitude of Low Frequency Fluctuation (fALFF)

fALFF represents the power within the low frequency range (0.01–0.08 Hz), divided by the total power in the entire detectable frequency range [30]. After the extraction of power spectra via a Fast Fourier Transform, the sum of frequencies in the low frequency band was calculated; the averaged square root of the power in the low frequency window, normalized by the mean within-brain values was obtained, and subsequently scaled by total power across all available frequencies [48].

### Degree Centrality (DC)

For each subject, each whole-brain voxel's time-series was extracted to calculate a temporal correlation matrix. We computed an individual Degree Centrality mask starting from study-specific functional volume mask, considering only voxels (in MNI 152 standard space) present in at least 95% of the participants, and further constrained by the MNI 152 25% gray-matter probability mask (r>0.25). Before graph definition, EPI time-series data were down-sampled to 4 mm isotropic voxel-size to reduce computational complexity. Then, voxel-based graphs were generated for each individual. Each voxel was considered as a node in the graph, and each significant functional connection (Pearson correlation) between any pair of voxels is an edge. To obtain each subject's graph, the correlation between the time-series of each voxel with every other voxel in the study mask was calculated (correlation matrix). For each subject, based on the graph, DC was calculated by counting the number of significant correlations between each voxel and all other voxels. DC indices were then transformed to z-scores based on each individual mean and standard deviation for DC across all voxels [29], [33], [49].

### Biological Parametric Mapping approach

The influence of gray matter (GM) atrophy in functional MRI analysis and consequently the regression of GM values as nuisance covariate is still a matter of debate [50]–[52]. Even if mean gray matter density is the more common approach to take into account cortical atrophy, this measure cannot consider regional differences in cortical atrophy. This is especially of interest in neurodegenerative diseases like FTD-*GRN*+, where cortical atrophy is not equally distributed between the anterior and posterior part of the brain and even between the two hemispheres. For this purpose, we adopted the Biological Parametric Mapping with Robust Regression (http://www.nitrc.org/projects/rbpm) approach [53], [54] (as toolbox of SPM8) that allowed a multimodal integrative imaging analysis, using resting-state fMRI data (ReHo, fALFF and DC maps, respectively) as the primary modality, and the corresponding voxel-based morphometry (VBM) data as imaging covariates (BPM ANOVA). We evaluated the effect of structural differences on fMRI data in a voxel-wise setting, using gray matter maps of each subject as a regressor of structural damage [55], [56]. We considered age and gender as covariates of no interest in the ANCOVA model. We applied this approach comparing FTD-*GRN*+ patients and related controls, as in a*GRN*+ no VBM abnormalities have been demonstrated [14], [17].

### Statistical analysis

SPSS package (v. 17.0, Chicago, IL, USA) was employed to run statistics for group differences in demographic andclinical characteristics and laboratory measures. Group comparisons were assessed by Mann-Whitney test or χ^2^ test, setting the statistical threshold to *P*-values Bonferroni's corrected ≤0.05.

For resting state fMRI analyses, the statistical significance was defined at the cluster-level using the non-stationary random field theory [57]. The A threshold of a p<0.01 uncorrected (cluster-forming threshold) allowed the identification of spatially contiguous voxels [58], [59]; then, false-discovery rate (FDR) correction (cluster-level, p = 0.05) was applied, and only surviving clusters were considered. To evaluate differences between groups (*aGRN+* vs yHC and *GRN+* vs HC), T-test was applied. Taking into account literature data on age-related changes in resting state fMRI functional connectivity [60]–[62], we studied the effect of age on connectivity measures considering the whole group of *GRN* carriers (both *aGRN+* carriers and FTD-*GRN+* patients) compared to healthy groups (young and old healthy controls). Difference of slope analysis was used to study the statistical interaction between age (as continuous variable) and genetic status (presence of *GRN* mutation), considering gender and clinical phenotype (1 =  asy-*GRN+*; 2 = FTD-*GRN+*; 3 = young HC; 4 = old HC) as nuisance variables, as previously applied by Garibotto et al. to evaluate cognitive reserve in Alzheimer's Disease [63]. Age regression analysis in GRN-mutation carriers and controls was performed and the statistical difference between the two regressions calculated (difference of slope analysis). For this analysis, two statistical thresholds were used: 1) p<0.01 uncorrected, 0.05 FDR cluster-level; 2) p<0.001 uncorrected, 0.05 FWE cluster-level, to evaluate the most significant clusters for each functional metrics applied (ReHo, fALFF, DC).

## Results

### Subjects

As shown in **Table 1**, there were no statistical differences between FTD-*GRN+* and age matched healthy controls (HC), regarding age or gender (for neuropsychological tests of FTD-*GRN+* see Supplementary Data). Considering asymptomatic subjects, *aGRN+* were older than their siblings not carrying GRN mutation (yHC, p = 0.05).

**Table 1:** Clinical and demographic characteristics of included subjects.

Variable	*FTD-GRN+*	*Healthy Controls*	*aGRN+*	*Young Healthy Controls*
60.4±5.3	n = 14	n = 16	n = 17	n = 22
Age at evaluation, y	60.4±5.3	59.7±8.7	41.6±9.0*	36.2±6.8*
Age at onset, y	58.8±6.2	-	-	-
Disease duration, y	1.93±1.9	-	-	-
Gender, female % (n)	64.3 (9)	75.0 (12)	47.1 (8)	63.6 (14)
Family history, positive % (n)	85.7 (12)	-	100 (17)	-
Clinical phenotype, bvFTD %(n)	50.0 (7)	-	-	-
FTD-CDR∧	6.0±3.5	-	-	-

FTD: Frontotemporal dementia; FTD-*GRN*+: FTD patients carrying Granulin Thr272fs mutation; a*GRN*+:

asymptomatic subjects carrying Granulin Thr272fs mutation; FTD-CDR: Frontotemporal Dementia modified Clinical Dementia Rating scale. * T-test comparison a*GRN*+ vs. young healthy controls p<0.05.

### Multimodal brain connectivity

**Figure 1:**
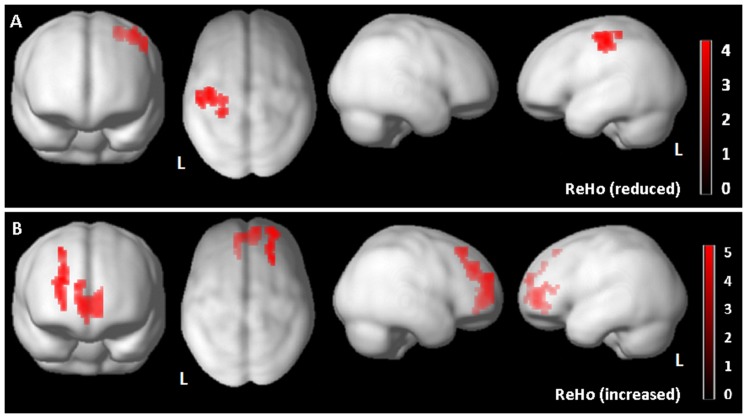
Regional Homogeneity (ReHo) Analysis in Asymptomatic *GRN+* carriers (*aGRN+*) compared to young healthy controls (yHC), showing reduced ReHo index (Panel A) as well as increased ReHo index (Panel B). The results are superimposed on a 3D-standarized T1 brain template. P<0.01 uncorrected, p<0.05 FDR cluster-level. L =  left.

In *aGRN+* compared to yHC, reduced ReHo was found in left parietal region (**Figure 1A** **,** **Table 2**). Furthermore, an increased ReHo index in mesial frontal cortex bilaterally, was evident(**Figure 1B** **,** **Table 2**), comparing *aGRN+* to yHC. No significant abnormalities of DC and fALFF indexes were reported.

**Table 2:** Alterations of brain connectivity parameters in asymptomatic *GRN* carriers as compared to healthy controls.

**Region ReHo (reduced)**	**x**	**y**	**z**	**T**	**P**	**Cluster size**
L Postcentral Gyrus	−27	−36	60	3.90	0.04	145
**Region ReHo (increased)**	**x**	**y**	**z**	**T**	**P**	**Cluster size**
R Superior Frontal Gyrus	24	39	33	4.75	0.05	119
R Medial Frontal Gyrus	6	54	3	4.10	0.01	189
L Medial Frontal Gyrus	−9	51	0	3.87	0.01	-

Talairach coordinates of significant voxels, at P<0.01 uncorrected, FDR-cluster level p<0.05. ReHo: Regional Homogeneity. R = right hemisphere; L  = left hemisphere.

**Figure 2:**
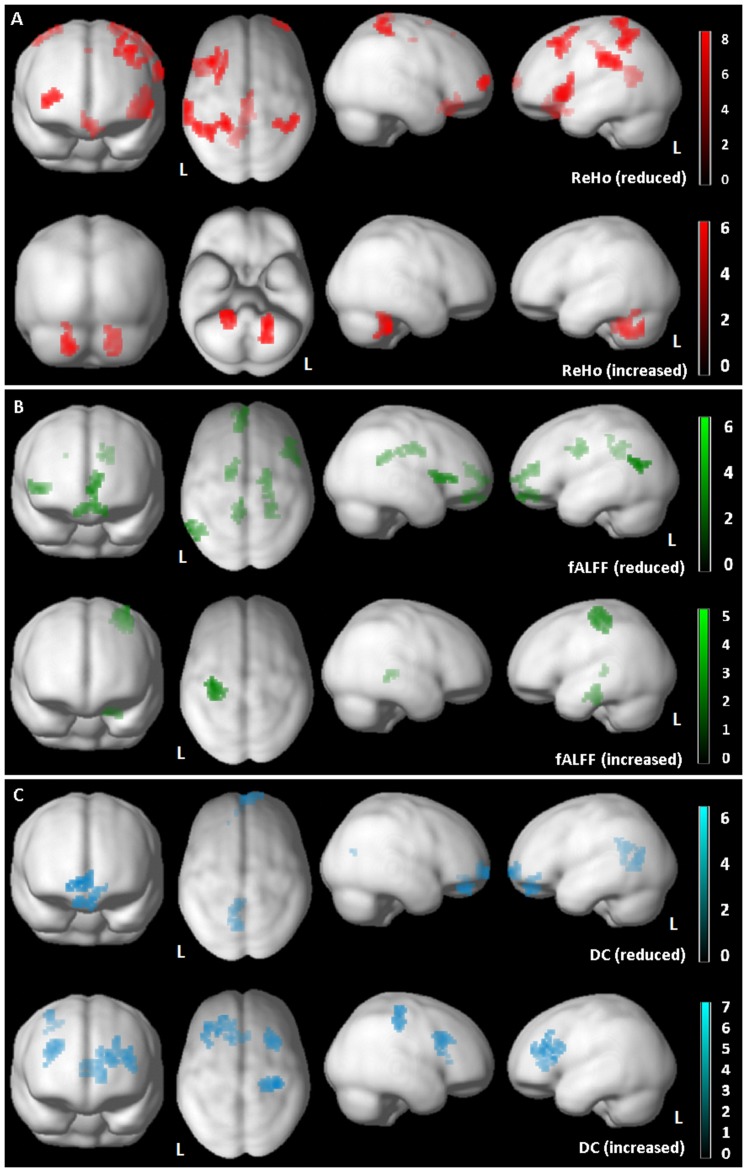
Regional Homogeneity (ReHo, Panel A), Fractional Amplitude of Low Frequency Fluctuations (fALFF, Panel B), Degree Centrality (DC, Panel C) analyses in Frontotemporal Dementia related to *GRN* (FTD-*GRN*+) compared to old healthy controls (oHC) using Biological Parametric Mapping Analysis (BPM) showing either reduced and increased significant clusters. The results are superimposed on a 3D-standarized T1 brain template. P<0.01 uncorrected, p<0.05 FDR cluster-level. L =  left.

FTD-*GRN*+ patients showed a reduced ReHo index in inferior parietal and frontal lobes, bilaterally, and posterior cingulate cortex as well as increased ReHo connectivity in cerebellar lobes, bilaterally (**Figure 2A** **,** **Table 3**).Furthermore, statistical analysis of fALFF showed a predominant impairment of frontotemporal regions in FTD-*GRN*+ patients with increased fALFF index in left precentral gyrus and hippocampal structures (**Figure 2B** **,** **Table 2**). Finally, FTD-*GRN*+ presented a reduced DC index in frontal pole bilaterally as well as in the posterior cingulate cortex. At the same time, increased DC index was evident in right postcentral regions and right dorsolateral prefrontal cortices, bilaterally (**Figure 2C** **,** **Table 2**). All the previous statistical analyses survived at the cluster-forming threshold of p = 0.01 uncorrected for multiple comparisons (FWE 0.05 cluster-level); no clusters survived at the more stringent threshold (cluster-forming threshold of p = 0.001 uncorrected for multiple comparisons (FWE 0.05 cluster-level)).

**Table 3:** Alterations of brain connectivity parameters in FTD-*GRN*+ as compared to healthy controls.

**Region ReHo (reduced)**	**x**	**y**	**z**	**T**	**P**	**Cluster size**
L Inferior Frontal Gyrus	−42	21	0	7.40	0.003	157
L Superior Frontal Gyrus	−39	24	51	6.86	0.013	117
L Inferior Parietal Lobule	−63	−27	36	6.43	0.015	103
L Medial Frontal Gyrus	0	27	−18	6.01	0.053	69
L Posterior Cingulate	−3	−30	24	5.14	0.001	194
**Region ReHo (increased)**	**x**	**y**	**z**	**T**	**P**	**Cluster size**
L Posterior Cerebellar Lobe	−21	−48	−33	6.03	0.02	126
R Posterior Cerebellar Lobe	18	−45	−39	5.18	0.02	106
**Region fALFF (reduced)**	**x**	**y**	**z**	**T**	**P**	**Cluster size**
L Superior Temporal Gyrus	−60	−63	18	5.84	0.024	66
R Anterior Cingulate Gyrus	21	−18	36	5.15	0.008	94
R Inferior Frontal Gyrus	45	27	3	5.09	0.024	65
L Posterior Cingulate	−6	−42	27	4.98	0.054	50
L Anterior Cingulate Gyrus	−18	−6	33	4.53	0.050	53
R Orbital Gyrus	9	45	−18	4.52	0.007	106
**Region fALFF (increased)**	**x**	**y**	**z**	**T**	**P**	**Cluster size**
L Postcentral Gyrus	−33	−33	60	5.55	0.003	124
L Parahippocampal Gyrus	−21	−24	−24	4.65	0.03	79
R Parahippocampal Gyrus	21	−36	−3	3.73	0.03	73
**Region DC (reduced)**	**x**	**y**	**z**	**T**	**P**	**Cluster size**
L Posterior Cingulate	−6	−66	15	5.40	0.026	61
R Medial Frontal Gyrus	12	66	−3	4.80	0.050	41
L Orbital Gyrus	−3	48	−21	4.31	0.026	53
**Region DC (increased)**	**x**	**y**	**z**	**T**	**P**	**Cluster size**
R Postcentral Gyrus	39	−27	45	6.34	0.005	74
R Middle Frontal Gyrus	27	18	33	4.98	0.005	72
R Anterior Cingulate Gyrus	6	21	15	4.47	<0.001	155
L Superior Frontal Gyrus	−21	45	21	4.39	<0.001	-

Talairach coordinates of significant voxels, at P<0.01 uncorrected, FDR-cluster level p<0.05. ReHo: Regional Homogeneity; fALFF: Fractional Amplitude of Low Frequency Fluctuation; DC: Degree Centrality. R = right hemisphere; L = left hemisphere.

### Effect of age on brain connectivity measures in GRN mutation carriers (aGRN+ and FTD-GRN+) (difference of slope analysis)

**Figure 3:**
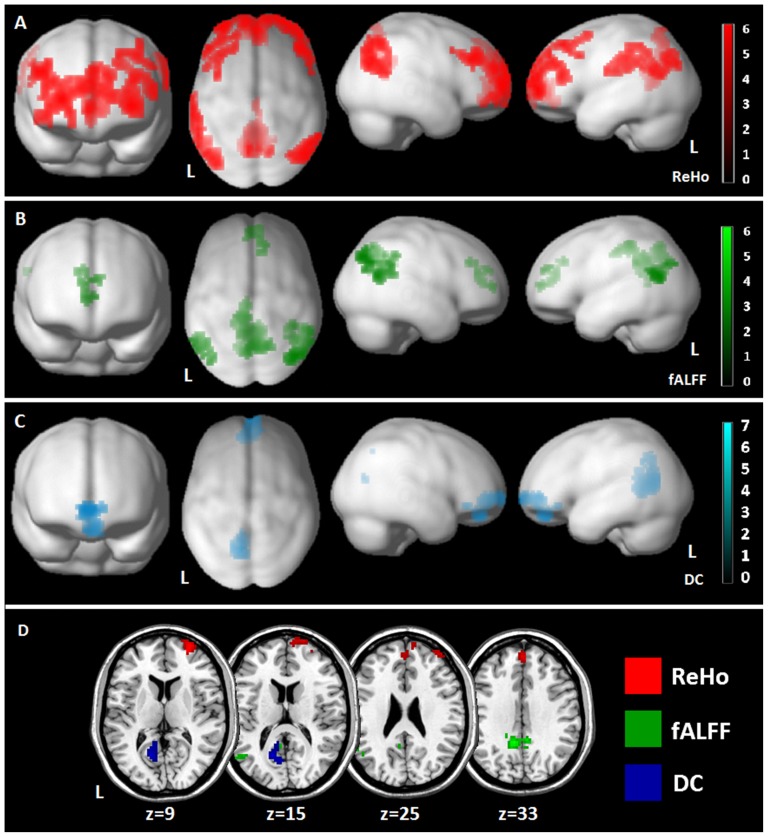
Effect of age on Regional Homogeneity (ReHo, Panel A), Fractional Amplitude of Low Frequency Fluctuations (fALFF, Panel B) and Degree Centrality (DC, Panel C) in *GRN*+ subjects (*aGRN*+ and FTD-*GRN*+) compared to healthy controls (yHC and oHC) (difference of slope analysis). The results are superimposed on a 3D-standarized T1 brain template. P<0.01 uncorrected, p<0.05 FDR cluster-level. L =  left. Panel D: presentation of all previous results at a more stringent threshold (p<0.001 uncorrected, p<0.05 FWE cluster-level), superimposed on an axial T1 brain template.

Considering all the subjects with *GRN* mutation (*aGRN*+ and FTD-*GRN*+) and healthy controls, a difference of slope analysis was applied to study the effect of age on ReHo, fALFF, and DC values. In ReHo analysis, an inverse correlation with age in *GRN*+ carriers compared to HC was evident, with reduced ReHo values in posterior cingulate cortex,inferior parietal lobule bilaterally, and anterior frontal regions (**Figure 3** **, panel A;** **Table 4**). Considering fALFF (**Figure 3** **, panel B;** **Table 4**), comparable results were found. DC showed an inverse correlation with age in orbitofrontal and posterior cingulate regions (**Figure 3** **, panel C;** **Table 4**). At a more stringent statistical threshold (p 0.001 uncorrected, FWE 0.05 cluster-level), only the following clusters survived: (ReHo analysis): right frontal region (x,y,z: 30, 63, 9; T = 5.26, 238 voxels, P = 0.006; R middle frontal gyrus, BA 10); (fALFF analysis): left precuneus (x,y,z: −9, −48, 33; T = 5.32, 138 voxels, P = <0.001; L precuneus, BA 31) and left superior temporal gyrus (,y,z: −57, −57, 21; T = 4.30, 45 voxels, P = 0.011; L superior temporal gyrus, BA 39); (DC analysis): left posterior cingulate region (x,y,z: −15, −63, 12; T = 6.61, 113 voxels, <P = 0.001; L posterior cingulate cortex, BA 30) (**Figure 3** **, Panel D**).

**Table 4:** Effect of age on multimodal connectivity measures in *GRN*+ subjects (*aGRN*+ and FTD-*GRN*+) compared to healthy controls (difference of slope analysis).

**Region ReHo**	**x**	**y**	**z**	**T**	**P**	**Cluster size**
L Superior Frontal Gyrus	−33	57	0	5.75	<0.001	1611
R Middle Frontal Gyrus	48	33	33	5.50	<0.001	1611
R Middle Frontal Gyrus	30	63	9	5.26	-	-
R Superior Frontal Gyrus	3	51	30	4.71	-	-
R Anterior Cingulate Gyrus	6	54	−3	4.11	-	-
R Middle Temporal Gyrus	51	−72	27	5.09	0.054	386
R Inferior Parietal Lobule	54	−60	45	4.98	-	-
L Posterior Cingulate	−15	−66	18	4.65	0.009	663
L Precuneus	−9	−51	27	4.45	-	-
L Inferior Parietal Lobule	−66	−30	33	4.40	0.034	465
L Postcentral Gyrus	−66	−18	21	4.22	-	-
**Region fALFF**	**x**	**y**	**z**	**T**	**P**	**Cluster size**
L Precuneus	−9	−48	33	5.31	<0.001	688
R Posterior Cingulate Gyrus	6	−45	33	4.60	-	-
L Paracentral Lobule	−15	−36	48	4.59	-	-
R Inferior Parietal Lobule	42	−72	39	2.94	<0.001	224
R Supramarginal Gyrus	39	−42	−39	3.85	-	-
L Superior Temporal Gyrus	−57	−57	21	4.30	0.005	138
R Anterior Cingulate Gyrus	12	33	30	3.65	0.036	86
**Region DC**	**x**	**y**	**z**	**T**	**P**	**Cluster size**
L Posterior Cingulate	−15	−63	12	6.61	0.002	286
L Precuneus	−9	−69	18	4.94	-	-
L Orbital Gyrus	−3	45	−21	3.98	0.004	224
R Superior Frontal Gyrus	9	57	−3	3.28	-	-

Talairach coordinates of significant voxels, at P<0.01 uncorrected, FDR-cluster level p<0.05. ReHo: Regional Homogeneity; fALFF: Fractional Amplitude of Low Frequency Fluctuation; DC: Degree Centrality. R = right hemisphere; L  = left hemisphere.

## Discussion

In recent years, great efforts have been made in the field of neurodegenerative diseases to fill the gap between molecular dysfunction at neuronal level and the macroscopic pattern of brain alterations. The application of advanced neuroimaging techniques especially to the monogenic dementias could represent the ideal model to test the natural history of proteinopathies and how they translate to clinical phenotypes [64]. Recently, a work on monogenic Alzheimer's Disease showed that decreased brain connectivity is already detectable in asymptomatic mutation carriers in those regions typically affected by the disease, with progressive damage as age increases [65].

If in Alzheimer's disease the temporo-parietal lobes are primarily affected, the involvement of frontotemporal areas in FTLD is largely proved. However, greater heterogeneity has been reported in the latter disease, depending on genetic traits and neuropathological features. As already reported, GRN mutations present asymmetric fronto-parieto-temporal atrophy, with the selective involvement of long intrahemispheric pathways [12], [13], [18], [66], mirroring the neuropathological distribution of TDP-43 type A proteinopathy [67]. A number of studies have corroborated these findings, demonstrating resting-state functional connectivity abnormalities since preclinical stages [13], [14], [16], [17], [68].

In the present work, we aimed to corroborate the disease signature by multimodal rs-fMRI and to assess the natural course of GRN-disease.

Altogether, these data suggest that the earliest feature of FTD associated with *GRN* mutations is the functional impairment of parietal lobes. Furthermore, looking at the increased functional connectivity in frontal regions, a potential compensatory mechanism cannot be excluded *a priori* [54], [69], as previously reported by our group [16].When disease progresses, as demonstrated by data obtained in FTD carrying *GRN* mutation and by regression analysis with age, more anterior regions, i.e. frontal lobes, are also involved. The explanation of increased connectivity in FTD-*GRN*+ patients is challenging, with the presence of regions not usually affected by the pathological process (i.e. cerebellar lobes) as well as regions surrounding the most involved parts of frontal lobes. Even if the role of hyperactivation in resting state fMRI is not completely defined, it is possible that regions not primarily involved, as well as regions in the less affected hemisphere or in the neighborhood of damaged areas could counteract functional impairment [54], [69], [70]. Taken together these results support the concept of fronto-parietal dementia, calling for a new nosology of the disease related to *GRN* mutation. In association with our previous work on asymptomatic *GRN* carriers [16], showing a reduced frontoparietal network connectivity as well an enhanced executive network connectivity compared to controls the present work support the idea that *GRN* related disease is characterized by an asymmetric (functional and structural) impairment via disconnection mechanism involving long white matter bundles connecting anterior/posterior regions, although the local connectivity approach cannot completely captures the functional alterations of long-range resting state brain networks. It is likely that the damage of parietal networks may not be identified by clinical evaluation and the diagnosis of FTD is made only when more detectable behavioral and cognitive disturbances depending on frontal dysfunction are overt.

Indeed, we observed a concordance of data by ReHo, fALFF and DC, but the former measure showed the higher sensibility in detecting functional alteration in the preclinical phase. ReHo analysis presented the highest test-retest reliability as compared to the other methods [46], [71]; furthermore, intra-regional perspective of Regional Homogeneity analysis captured local dynamics of network functioning, as a basis for the subsequent broad alterations of the entire network [54], [72]–[74].

Clearly defining the functional alterations sustaining *GRN* pathology is crucial both for a better understanding of the disease, and in order to define biomarkers to test disease-modifying drugs against TDP43 proteinopathies. In this view, Warren and colleagues have recently proposed the term of “molecular nexopathies” as a theoretical paradigm that different proteinopathies (i.e., TDP-43 or MAPT) could differently affect neural networks [66], [75].

Our work presents some limitations. First, the described model of disease progression derives from a cross-sectional approach, and longitudinal studies with structural and functional imaging are needed to confirm our hypothesis. Second, in FTD patients we applied a voxel-by-voxel covariance of gray matter density, to take into account the presence of focal pattern of brain atrophy (Biological Parametric Mapping) [53]–[55], but the role of GM correction is still an open question. Third, the small sample size (14 FTD-*GRN*+patients (7 bvFTD and 7 avPPA) did not allow a subanalysis for each clinical phenotype for neuroimaging and clinical/neuropsychological data.

In conclusion, our work extends previous findings on the brain functional correlates of *GRN* related pathology by multimodal rs-fMRI approach. The prevalent posterior functional impairment in the preclinical stage, with a progressive involvement of frontal regions during the course of the disease, moving from the preclinical to the clinical onset of the disease, suggests that the term of frontoparietal dementia for *GRN* related disease may be considered.
